# 42 Burnout in the Burn Unit: An Analysis of Surgeon Wellness

**DOI:** 10.1093/jbcr/iraf019.042

**Published:** 2025-04-01

**Authors:** Anastasiya Ivanko, Jonathan Schoen, Herbert Phelan, Jeffrey Carter

**Affiliations:** Burn Center at University Medical Center; Louisiana State University Health Sciences Center - New Orleans; Louisiana State University Burn Center; Louisiana State University Burn Center

## Abstract

**Introduction:**

The United States is a facing a surgeon shortage with a projected shortage of over 29,000 surgeons by 2030. This workforce crisis could result in fewer burn care specialists as surgeons opt for competing specialties to which they receive greater exposure in training. With only 0.4% of the surgeon workforce being burn surgeons, the loss of additional recruits to the field could result in a critical reduction in available and qualified burn care. To recruit and retain burn surgeons, it’s essential to understand their stressors with workload benchmarks that consider the complexity and strain associated with providing operative burn care.

**Methods:**

After obtaining Institutional Review Board (IRB) approval, we conducted a de-identified survey regarding burn surgeon wellness to gain insights into their well-being. Solicitation to participate and data acquisition was done by an American Burn Association (ABA) email and Survey Monkey™. Continuous and dichotomous variables were analyzed using Microsoft® Excel.

**Results:**

We received 77 responses from burn surgeons (37.6% female, 62.3% male). Most (95.0%) take burn call and perform surgery, with 79.5% in academic settings. While 73.4% are satisfied with their career and 91.0% appreciate the complexities of burn care, 54.0% feel they lack time for personal activities, and 61.8% didn’t take enough time off for childbirth. Additionally, 39.7% experience burnout, and 25.6% consider leaving the field. The majority (54.4%) feel they lack time to recover after demanding cases, and 65% believe burn surgery negatively impacts their health. Job related health impacts include musculoskeletal issues (66%), skin conditions (29.5%), and sleep deprivation (89.7%). Despite these challenges, they report taking only one sick day annually. Currently their ability to meet the demands of the job is rated at 7 (±1.1), yet they foresee a decline over the next two years, with a prognosis averaging 4.2 (±0.5).

**Conclusions:**

Our study reveals substantial challenges facing burn surgeons, despite their overall satisfaction with the profession. The prevalence of burnout symptoms, consideration of leaving the field, and the impact on personal and family well-being underscore the need for interventions to support their mental and physical health. Proactive interventions and further research are needed to create a supportive environment that enables burn surgeons to thrive professionally while safeguarding their quality of life.

**Applicability of Research to Practice:**

1. The physical ailments reported by burn surgeons highlight the need for workplace safety protocols to reduce strain and promote a healthier environment.

2. Recognizing the projected shortage of burn surgeons enables healthcare institutions and policymakers to develop strategies for recruitment and retention, ensuring adequate specialty car

**Funding for the Study:**

N/A

